# The Needs of Adolescents to Communicate with Their Parents in Online and Offline Formats: The Creation and Validation of a Questionnaire

**DOI:** 10.11621/pir.2024.0102

**Published:** 2024-03-01

**Authors:** Ilia A. Larin, Tatiana Yu. Sadovnikova

**Affiliations:** a Lomonosov Moscow State University, Russia

**Keywords:** Adolescence, child-parent relationships, communication, digital communication, communicative needs of adolescents, offline and online communication

## Abstract

**Background.:**

Modern society is characterized by the widespread use of social media, which provides users with new communication, leisure, work, and study opportunities. The growth of online communication time leads to the development of new special communicative needs. These circumstances prompted us to develop the “Questionnaire on Adolescents’ Needs to Communicate with Their Parents Online and Offline” (QANCP).

**Objective.:**

We set out to study the relationship between adolescents’ hierarchy of communicative needs and the characteristics of child-parent relations by developing and testing the psychometric characteristics of the QANCP.

**Design.:**

One hundred and twenty-eight teenagers (56 boys and 72 girls, age 15–17) took part in the research. The research methods included theoretical analysis, modeling, questionnaires, and statistical analysis. The tools used were: 1) the customized (authorial) “Questionnaire of adolescents’ needs to communicate with their parents online and offline” (QANCP); and 2) the Inventory of Parent and Peer Attachment (IPPA) by Armsden and Greenberg (1987) in the Sadovnikova and Eliseeva adaptation (Eliseeva, 2013).

**Results.:**

The results of the qualitative research proved the acceptability of the QANCP scales. Expert assessments showed that the test had sufficient face and content validity. Statistically significant differences in the parameters measuring adolescents’ communicative needs in groups with different types of relations with their parents were established.

**Conclusion.:**

Testing of the QANCP questionnaire showed that the QANCP is a valid and reliable instrument to measure adolescents’ communication with their parents in online and offline formats. It was established that there is a connection between the characteristics of child-parent relations in adolescence and the hierarchy of communication needs of “adolescent-parent” communication online and offline.

## Introduction

Adolescence is known as the key period in personality development (Elkonin; Erikson; Rice; Stern; Vygotsky). The child’s increased intelligence and socio-moral capabilities lead to intensive changes in all spheres of personal development (Craig, 2001; Molchanov, 2023; Tolstykh, 2017). Transforming the relationship between children and their parents is a prerequisite for teenagers to grow up (Tolstykh, 2019). Nowadays this transformation takes place under the conditions of the Digital Era.

### The Phenomenon of the “Digital socialization”

According to the latest studies, we can’t discuss modern teenagers’ developmental situation without considering the environment in which this development takes place. Information and computer technologies (ICT), which constitute the “technosphere” of modern human development, directly affect human interaction with the world (Otrel-Cass, 2019; Soldatova, & Rasskazova, 2020). In this regard, we can talk about the convergence of the realities of “online” and “offline” for a modern person, which creates completely new conditions for the development of modern adolescents (Soldatova, & Voiskunskii, 2021; Speicher et al., 2019). It is notable that ICT combine instrumental and iconic components that are key to human development. According to the theory of L.S. Vygotsky, the Internet, telecommunications, and IT tools are the habitat of generation Z, making them the cultural and historical tools that contribute to the generation of new forms of activity, cultural practices, phenomena, and meanings (Rubtsova, 2019).

Thus, modern teenagers experience reality as “mixed,” without a clear division between online and offline. This leads to such phenomena as hyper-connection, which is expressed in more screen time, an expanded personality, and digital sociality acquired during interpersonal communication on the Internet, among many other things (Brubaker, 2020; Otrel-Cass, 2019; Soldatova, & Rasskazova, 2020; Soldatova, & Voiskunskii, 2021). A new phenomenon is emerging: the phenomenon of “digital socialization,” which affects the development of modern adolescents and their communication with other people, in particular their parents.

Digital socialization is a generally recognized condition for children’s mental development (Abramenkova, 2020; Martsinkovskaia, 2019). Research on the Internet and its role in the lives of children, adolescents, and adults has led to the clarification of a wide range of concepts related to the problem of personality development in the context of ICT penetration into all areas of life (Voiskunskii, & Soldatova, 2019; Wickersham, et al., 2021). The Internet is seen by modern researchers as a new space for socializing children and adolescents, which offers both opportunity and risk. Among the most discussed problems are cyberbullying; the possibilities and restrictions of parents controlling information content; the attractiveness for children of computer games, network information, etc.; excessive involvement of some children and adolescents in virtual interactions; emotional disadvantage; and behavioral disorders (Clayborne et al., 2019; Guntuku et al., 2017; Polskaia, & Yakubovskaya, 2019; Voiskunskii, & Soldatova, 2019).

Traditional and digital socialization are complementary and mutually dependent processes. This process is experienced differently by children and parents. Intergenerational relationships within families and kinship have become a salient issue in scientific research (Pramono et al., 2019). It is noted that the virtual world is more significant for teenagers than for their parents; modern teenagers have a mixed picture of the world to a greater extent than their parents do, where online does not displace, but complements offline. Parents, especially those with a low level of digital competence, have a more negative assessment of the impact of the online environment on their children’s development. However, it is worth noting that, for both teenagers and parents, interpersonal interaction in the real world continues to play a much greater role than digital communication (Soldatova et al., 2022).

Modern world challenges highlight the crucial importance of young people developing new skills, especially digital literacy (Karabanova, & Georgievskaia 2019; Moore, et al., 2011; Tomczyk, & Eger, 2020). Communicative competence -- the ability to flexibly and effectively use the digital environment in communication in a family, educational, and working context -- is included in the list of the most important guidelines for the development of the modern personality.

The presence of smartphones in modern adolescents’ lives is estimated as close to 100%. Domestic and foreign studies show an insuffcient level of digital competence in both children and their parents (Mossberger, et al., 2008; Soldatova et al., 2022).

As we know, in Russia’s large cities (for example, in Moscow), only 1-2% of all schoolchildren do not use the Internet (Regush et al., 2023). It is noted that nowadays teenagers devote more time to smartphones and computers than to their own social and emotional development. In the United States, 95% of teenagers have access to smartphones, and 45% of teenagers stay online permanently (Anderson, & Jiang, 2018; Pramono et al., 2019). Russian teenagers have one of the highest levels of screen time in Europe on weekdays and a slightly above average level on weekends. The content of Russian teenagers’ online activities is more diverse than that of their peers from Europe (Soldatova, & Rasskazova, 2023).

### Teenagers’ communication with parents and peers in digital word: the new data

During adolescence, communication with peers and parents is influenced by the social context of a child’s development (L.S. Vygotsky); teenagers’ usage of digital devices is a part of that developmental context (Branje, 2018).

The scientific study of communication has a long history. Various theories and models of communication have been proposed (McQuail, & Windahl, 2013). According to E.P. Il’in (2012), the need for communication raises two important issues about its origins. The first is whether the need for communication (or communicative need) is a specific need different from other social or spiritual needs, or whether it is one variety of them. Secondly, if a separate need for communication does exist, then what is its origin: is it innate (basic) or secondary? That is, is it formed in ontogeny or in the process of socializing the child? Both of these issues were the subject of consideration in the monograph of M.I. Lisina (1986). Most domestic scientists (N.F. Dobrynin, A.G. Kovalev, A.V. Petrovsky, B.F. Parygin, and K. Obukhovsky) believe that the need for communication is a specific independent human need, different from other needs. There is a similar point of view expressed in the works of foreign psychologists (M. Ainsworth).

However, in practice, the need for communication is often reduced to more private needs: the need for making an impression (M. Kistyakovskaya), the need for security (A. Peyper), and the need for comfort from contact with a soft warm body (H. Harlow, & M. Harlow). We consider the position of L.I. Marisova (1978) to be the most logical. L.I. Marisova proposed a model of a hierarchical structure of communicative needs, which serves as the motivational basis for communication. In her work, nine groups of communicative needs were identified (Marisova, 1978).

It is interesting to view communication on the Internet from the point of view of the communication model proposed by G.M. Andreeva (Andreeva, 2001). When studying communication in the Internet environment, it is possible to trace transformations of the communicative, perceptual, and interactive sides of communication. The model by G.M. Andreeva determined the theoretical framework for our study (Larin, & Sadovnikova, 2020, 2023), part of which we present in this publication.

The risks associated with the transformation of the communicative side of online communication are noted. The absence of nonverbal signals in communication is a serious obstacle to the exchange of information, which can lead to misinterpretation of the message and the emergence and aggravation of conflicts (Walther, & Parks, 2002).

Some researchers talk about a strong relaxation of social norms when communicating on the Internet, while others, on the contrary, note their restriction (Molchanov et al., 2019). The characteristics of the communicative side of communication vary and are transformed to a large extent in the Internet space.

It is noted that there is a connection between the level of moral consciousness and the willingness of young Internet users to comply with the basic norms in online communication (Karabanova, & Georgievskaia, 2019; Molchanov, et al., 2019). Internet users with a high level of moral competence are more likely to adhere to the basic ethical norms.

Interaction in the Internet space, expressed in the interactive side of communication, mainly includes a sociological or political aspect: people make new acquaintances, and unite in thematic communities (Voiskunskii, 2016).

A number of studies have been devoted to the problem of the transformation of the friendship phenomenon during online communication (Pogorelov, & Rylskaia, 2022; Rubtsova, et al., 2021; Twenge, 2019). Teenagers distinguish positive and negative sides of online communication with peers. The positive ones include: the ability to communicate at a distance, as well as the opportunity to socialize and get acquainted. Among the negative sides are: the possibility of building a false image, the diffculty in understanding individual messages, and the addictive nature of online communication (Rubtsova et al., 2021).

Teenagers’ communication in the Internet environment depends on their experience in real life. Behavioral patterns on the Internet turn out to be interrelated with past life experiences (Karabanova, & Georgievskaia, 2019; Molchanov, 2023).

Transformations related to the perceptual side of communication are of great interest. Research (Belinskaia, & Gavrichenko, 2018; Soldatova et al., 2022; Zimmermann et al., 2022), has shown that during communication in the Internet space, we form a virtual or alternative identity. An alternative identity occurs when a user of social networks creates more than one profile. Accordingly, the position taken by him in the process of communication, his perception of other people, and how other people react to him, changes.

The Internet space and computer technology, in turn, provide limitless opportunities for the realization of individual human freedom (Karabanova, & Georgievskaia, 2019).

Among the factors influencing a teenager’s desire to create an alternative identity are such characteristics of online communication as the decrease in visual and auditory stimuli that push for a new presentation of his physical self (Vakelenburg et al., 2005). Anonymity in the Internet space, which avoids consequences possible in “real” life, also serves as a motivation for teenagers to build an alternative identity. The possibility of experimenting with one’s identity (which once again addresses the issues of self-determination) stands out as the main motive for building an alternative identity (Vakelenburg et al., 2005).

Family communication is influenced by all the digital communication phenomena described above. Thus, family communication continues to play a key role in human development, but inevitably is transformed. Our research is devoted to the study of this transformation (through the prism of the communicative needs of adolescents).

## Methods

We set out to study the phenomenon of online and offline communication between teenagers and their parents.

The research questions were: A) Are the needs of adolescents to communicate with their parents the same or different in online and offline formats are the same or different; and B) How are the needs of adolescents to communicate with their parents in online and offline formats related to the type of child-parent relationship.

### Participants

The participants were 128 pupils from 10th-11th grades in Moscow schools, ranging in age from 15 to 17; among them were 33 15-year-olds (26%); 34 16-year-olds (27%); and 61 17-year-olds (47%). There were 72 girls (56.0 %) and 56 boys (44.0 %) who participated in the quantitative part of the presented study.

### Procedure

The qualitative research consisted of two parts. The first was the development and testing of the authorial “Questionnaire of adolescents’ needs to communicate with their parents online and offline**”** (QANCP). The second part was focused on the relationship between type of child-parent relationship and the needs of the adolescents to communicate with their parents.

The participants completed the authorial questionnaire (QANCP) and the Russian version of the Inventory of Parent and Peer Attachment (IPPA) by Armsden and Greenberg (1987) in the T.Yu. Sadovnikova and O.V. Eliseeva adaptation (2013).

The research was conducted on the basis of voluntary participation, anonymity, and confidentiality. The adolescents were informed about the study protocol beforehand. They were given the opportunity to meet individually to discuss their individual results. The research was conducted by I.A. Larin, who worked as school psychologist and had an extensive practice of counseling adolescents.

### Questionnaires

#### Questionnaire on adolescents’ needs to communicate with their parents online and offline (QANCP) by I. Larin & T. Sadovnikova (Larin, & Sadovnikova, 2020)

The QANCP is composed of two main scales to identify adolescents’ needs to communicate with their parents in online and offline. Each QANCP scale includes nine subscales characterizing the communicative needs of adolescents, as highlighted in the classification by L.I. Marisova (Marisova, 1978):

1) the need for another person and the need for relationships with another person (RPer);
2) the need for belonging to social community (BCom);
3) the need for co-experience and empathy (Emp);
4) the need for care, assistance, and support from others (Sup);
5) the need for providing assistance, care, and support to others (PrSup);
6) the need for establishment of business links for joint activities and cooperation (JAct);
7) the need for constant exchange of experience, knowledge (Exch);
8) the need for assessment by others, respect, authority (Resp);
8) the need for developing an understanding and explanation of the objective situation and everything that happens in it (In Com) in common with other people.

These nine basic communicative needs served as the basis of the questionnaire. Since the questionnaire was aimed at a narrow area -- the study of data on the need for communication between parents and children in online and offline formats — we presented two scales (communicative needs online and communicative needs offline), each of which included the nine subscales. (See the Appendix for the texts of the two scales)

Each sub-scale of the QANCP included two statements. The QANCP thus contained 36 items. The teen’s assessment was made on a 5-point Likert scale, where 1= no; 2 = probably not; 3 = I don’t know; 4 = probably yes; and 5 = yes.

#### The Inventory of Parent and Peer Attachment (IPPA; Armsden & Greenberg, 1987)

The original version of the IPPA was developed to measure attachment in older adolescents. It assesses the positive and negative affective and cognitive dimensions of adolescents’ relationships with their parents and close friends.

In our study, the IPPA was used for studying research question B, which evaluated how the communicative needs of adolescents to communicate with their parents in online and offline formats related to the type of child-parental relationship. The IPPA was used as a valid and secure method reflecting the main aspects of the child-parental relationship in adolescence.

We utilized the latest version of the IPPA (Armsden & Greenberg, 1987), which comprises three different forms, one each for mother, father, and peer, and consists of 25 items (we used the mother and father forms). In each form, the item format was a five-point Likert scale: 5 = (almost always or always true); 1 = (almost never or never true). Each form yielded an overall score for attachment security, as well as three subscale scores: trust, communication, and alienation.

## Results

The results were statistically analyzed with the IBM SPSS program, ver. 21.0.

### Results of testing the psychometric characteristics of the QANCP questionnaire

#### The reliability measures

When testing the authors’ QANCP questionnaire (“Questionnaire of Parents and Adolescents Communication needs”) for the two main scales, we found that Cronbach’s α was .713 and the Spearman Brown coeffcient was .682 were obtained. These results indicated suffcient internal consistency and split reliability of the main scales of the QANCP questionnaire.

For the sub-scales characterizing the adolescents’ communication with their parents in the online and offline formats, Cronbach’s α values of .908 and .892 were obtained, respectively. The Spearman-Brown coeffcients for the two subscales were, respectively .815 and .869, suggesting high internal consistency and split reliability of the questionnaire subscales.

The Kolmogorov-Smirnov coeffcients we obtained for the subscales of the QA-NCP questionnaire were offline (p = .20 > 0.05; K-S = .067) and online (p = .20 > .05; K-S = .057); these results confirm the correspondence of the data distribution by sub-channels to the normal Gaussian distribution.

### Study of preferred forms of communication between adolescents and parents

In *Table 1* the intensity of the adolescents’ needs to communicate with their parents is presented. Each subscale (each communicative need) contained two statements. The assessment for each statement was made on a 5-point Likert scale.

**Table 1:** Intensity of communication needs of adolescents in communication with parents

	Communicative Need, “offline” format			Communicative Need, “online” format		
Communicative Need	Rank	Mean	SD	Rank	Mean	SD
The need for another person and the need for relationships with another person	2	7.5*	2.00	2	5.69*	2.48
The need for belonging to social community	3	7.47*	2.42	8	4.52*	2.60
The need for co-experience and empathy	9	6.33*	2.62	9	4.25*	2.47
The need for care, assistance, and support from others	5	7.16*	2.50	6	4.78*	2.65
The need for providing assistance, care, and support to others	6	7.03*	2.31	4	5.25*	2.70
The need for establishment of business links for joint activities and cooperation	1	7.52*	2.13	1	7.77*	2.51
The need for constant exchange of experience, knowledge	4	7.21*	2.26	5	4.81*	2.40
The need for assessment by others, respect, authority	8	6.89*	2.26	3	5.34*	2.46
The need for developing an understanding and explanation of the objective situation and everything that happens in it in common with other people.	7	7.01*	2.41	7	4.63*	2.42
General Need for Communication		64.11*	15.33		47.04*	17.24

Note. * = (Wilcoxon, p ≤ .05)

The intensity of all the adolescents’ needs to communicate with their parents differed significantly when they were communicating online and offline (Wilcoxon test, p = .000 < .05). In almost all cases, the needs expressed in the offline format were significantly more pronounced than similar indicators for the online format. The exception was the communicative need “to establish business ties for joint activities and cooperation” (“everyday motive”), where the intensity of the communicative need when communicating online was significantly higher than when communicating offline (7.77 > 7.52;).

The overall need of the adolescents to communicate with their parents in the offline format was also significantly higher than the need for communication with their parents in the online format (64.11 > 47.04).

The most pronounced motivations for offline communication were the communicative needs “for establishing business ties for joint activities and cooperation” (7.52), “for another person and a relationship with him” (7.50), and “for belonging to social community” (7.47).

The most pronounced motivations for online communication were the communicative needs “for establishing business ties for joint activities and cooperation” (7.77), “for another person and relationship with him” (5.69), and “for evaluation by others, authority” (5.34).

Thus, the “everyday motive” and the communicative need “for another person and for relationships with him” were the most pronounced for both forms of communication (offline and online). At the same time, the communicative need “to belong to the social community” was more pronounced for communication offline, and “for the assessment by others, authority” for communication online.

### Adolescent Child-Parent Relationship Study

**Figure 1.**
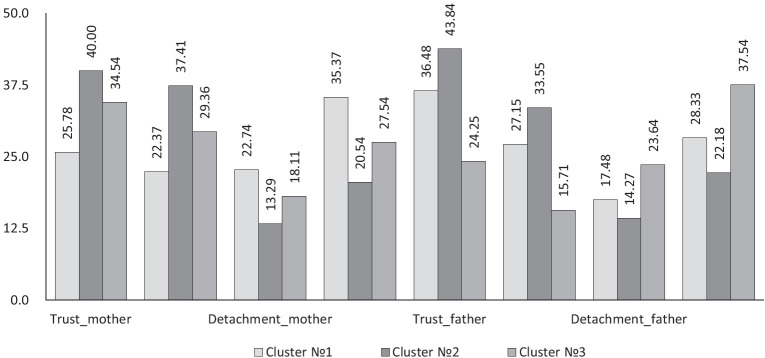
Distribution of children-parents relationship characteristics by clusters (IPPA)

A cluster analysis was carried out on the eight scales of the IPPA questionnaire. Depending on the characteristics of the child-parent relationship, three clusters (groups) were identified. Significant differences were established for all clustering scales by calculating the Kruskal-Wallis criterion (p = 0.000<0.05). Results are presented in *Figure 1*.)

*Group No.1 **(“trust in the father” — TF)*** included adolescents (24% of the sample) with high values on the scales of “detachment” and “attachment” to the mother, but low values on the scales of “communication” and “trust” in the mother, as well as with high values on the scales of “communication” and “trust” in the father and low values on the scales of “detachment” and “attachment” to the father. This group can be characterized as teenagers who have a more trusting and close relationship with their father.

*Group No. 2* (***“trust in both parents” — TP***) included adolescents (50% of the sample) with high values on the scales of “communication” and “trust” and low values on the scales of “detachment” and “attachment” to both father and mother. This group can be characterized as teenagers who have close, trusting, and reliable relationships with both parents.

*Group No.3 **(“trust in the mother” — TM)*** included adolescents (26% of the sample) with high values on the scales of “detachment” and “attachment” to the father, but low values on the scales of “communication” and “trust” in the father, as well as with high values on the scales of “communication” and “trust” in the mother and low values on the scales of “detachment” and “attachment” to the mother. This group can be characterized as teenagers who have a more trusting and close relationship with their mother.

### Comparative study of the intensity of communicative needs with parents of adolescents with different types of child-parent relations

We investigated the presence of significant differences in the intensity of certain needs reflected in communication between adolescents and their parents, as identified in the subscales of the QANCP, depending on the characteristics of child-parent relations (belonging to the selected clusters).

There were significant differences in the intensity of the overall need for adolescents to communicate with their parents offline (p = .000) and online (p = .011), as well as in the intensity of individual communicative needs of adolescents to communicate with their parents online and offline between the three groups (Kruskal-Wallis). Thus, we found significant differences in the intensity of adolescents’ needs to communicate with their parents depending on their child-parent relationship style (that were highlighted in clusters).

As for the teenagers’ needs to communicate with their parents offline, significant differences were revealed for all communicative needs (p = .000), except for the need to “establish business ties for joint activities and cooperation” (K-W = 0.297, p = .132).

For the adolescents’ need to communicate with their parents online, significant differences were identified for the needs: “for belonging to a social group” (K-W = 11.914, p = .003); “for co-experience and empathy” (K-W = 6.651, p = .036); “for caring, helping and supporting others” (K-W = 6.791, p = .034); “for helping, caring, and supporting others” (K-W = 7.366, p = .025); and “to develop a common understanding and explanation of the objective situation and everything that happens in it with other people” (K-W = 7.894, p = .019).

A comparison was made between the intensity of adolescents’ needs to communicate with their parents online and offline, depending on the group identified by the characteristics of the child-parent relationship. The results are in *Table 2*.

**Table 2:** Comparison of the intensity of communicative needs in communication of adolescents with parents online and offline with different types of children-parent relations

Communicative Need	TF			TP			TM		
	Mean offline	Mean online	p	Mean offline	Mean online	p	Mean offline	Mean online	p
The need for another person for relationships and the need with another person;	6.8	5.6	.012	8.4	6.0	.000	6.3	4.7	.001
The need for belonging to social community;	6.5	4.3	.000	8.5	5.3	.000	6.1	3.2	.000
The need for co-experience and empathy;	4.7	4.2	.164	7.2	4.7	.000	5.9	3.4	.000
The need for care, assistance, and support from others;	5.2	4.1	.010	8.5	5.4	.000	6.2	4.1	.000
The need for providing assistance, care and support to others;	5.8	5.1	.900	8.0	5.7	.000	6.0	4.0	.001
The need for establishment of business links for joint activities and cooperation;	7.4	7.8	.302	7.9	8.0	.645	7.1	7.9	.103
The need for constant exchange of experience, knowledge;	5.9	4.7	.009	8.4	5.2	.000	6.1	4.2	.000
The need for assessment by others, respect, authority;	5.6	4.9	.112	7.75	5.9	.000	6.2	4.7	.013
The need for developing an understanding and explanation of the objective situation and everything that happens in it in common with other people.	5.7	4.1	.002	8.14	5.4	.000	6.1	3.9	.001
General Need for Communication	53.6	44.9	.000	72.9	51.7	.000	56	40.2	.000

*Note. TF = trust in father. TP = Trust in parents. TM = trust in mother*

When analyzing the results obtained for the TP group, significant differences were revealed between the intensity of the needs to communicate with parents online and offline for all communicative needs (p = .000<.05), except for the communicative need for “establishing business ties for joint activities and cooperation” (W = –.461, p = .645>.05). The needs of adolescents to communicate with their parents offline are much bigger than online.

Similar results were obtained for the TM group, where no significant differences were found for the communicative need “to establish business ties for joint activities and cooperation” (p = .103>.05), unlike the others. The needs of adolescents to communicate with their parents offline are much bigger than online for this group too.

When analyzing the results obtained for the group TF, significant differences were revealed between the intensity of the needs to communicate with parents online and offline for a number of communicative needs: “for another person and relationships with him” (W = –2.516, p = .012 < .05); “for belonging to a social community” (W = –3.843, p = .000 < 0.05); “for the care, help, and support of others” (W = –2.584, p = .010 < .05); “for the constant exchange of experience, knowledge” (W = –2.596, p = .009 < .05); and “for developing a common understanding and explanation of the objective situation and everything that happens in it” (W = –3.159, p = .002 < .05). For each group with significant differences, the needs of the adolescents to communicate with their parents offline were much bigger than online.

When analyzing the results obtained for all three groups, significant differences were revealed between the intensity of the general need to communicate with parents offline and online:

- For TF (W = –3.591; p = .000 < .05; mean rank (offline) = 13.47 > mean rank (online) = 5).
- For TP (W = –5.963; p = .000 < .05; mean rank (offline) = 28.9 > mean rank (online) = 8.4).
- For TM (W = –4.49; p = .000 < .05; mean rank (offline) = 15.38 > mean rank (online) = 3).

## Discussion

1. The high intensity of the need “for evaluation by others, authority” when communicating with parents online is explained by the fact that, under modern conditions of the blurring of the boundaries of adolescence and the extended period of childhood, adolescents remain addicted to adult assessment for a longer period of time. Communication with parents online allows the adolescents to get this assessment at any convenient moment, and the mediation of ICT communication reduces the emotional tension felt by a teenager when assessing his personality with a close adult. We consider the use of ICT in communication with parents as a means (psychological instrument) of “mastering yourself,” as discussed by L. Vygotsky (Vygotsky, 2021). This task is solved by increasing the teenager’s ability for reflection, semantic generalization, self-regulation, and self-determination (Tolstykh, 2019; Vygotsky, 2021).

The need “for belonging to the social community” in the communication of adolescents with their parents offline is characteristic of this form of communication, and is expressed in the usual family rituals. These are emotionally important for adolescents as part of family life in a parental family and the attachment of adolescents and young men to their home, which was noted both by respondents in our focus group study and in scientific works.

2. Three groups of adolescents were identified that had qualitatively different relationships with their parents: 1) the first group had a trusting relationship with the father and were ambivalent about the mother; 2) the second had a trusting relationship with both parents; and 3) the third had a trusting relationship with the mother and were ambivalent about the father.

It is known that child-parent relations are the most important component of the SSD (the Social Situation of Development — Vygotsky, 2021)), and act as a fundamental precondition for the development of the child’s personality. The identified types of relationships with parents, which were significantly different in terms of trust, communication, and interpersonal distance, showed one of the mechanisms for the formation of variability in the trajectories of the modern adolescents’ personality formation. Our data correlate with recent studies (Kins et al., 2013).

3. Thus, when analyzing the groups TP and TM, it was clear that in almost all cases, the needs of adolescents to communicate with their parents offline were greater than online. The exception was the need “to establish business ties for joint activities and cooperation.” for which no significant differences were found in both groups, and higher values were obtained for online communication between adolescents and parents.

Perhaps the preference for the online format in communication with parents related to the organization of joint activity is determined by the rhythm of the daily life of modern adolescents and their parents. It is known that high school students devote a lot of time to their learning load. Teenagers in Moscow, as a rule, are additionally engaged in seeking successful admission to a selected university, which increases the level of stress in adolescents and youth, and leads to the risk of disorders in emotional development (Tikhomandritskaia, & Rikel, 2022; Tung, & Sandhu, 2005).

These results suggest that material and household topics are beginning to play one of the leading roles in the communication of modern older teenagers with their parents, and online communication is the most convenient method for its implementation. The findings reflect trends identified in a number of studies on the increased importance of the attachment of children and adults to the family home.

## Conclusion

Adolescents prefer direct forms of communication with their parents instead of online communication.

The personal nature of offline communication provides a greater opportunity for trust and engagement due to the ability to perceive the emotional component of communication.

A variety of motivations for accessing indirect communication between adolescents and parents (online format) was revealed: the most frequent was to solve everyday issues (business form of communication).

The possibilities of resolving conflict situations in the child-parent interaction arise as a result of the fact that use of online communication can reduce emotional tension.

The authorial QANCP questionnaire was tested. The conclusion was that the authors’ version of QANCP is a valid and reliable instrument to measure adolescents’ Communication with Parents in Online and Offline Formats.

It has been established that there is a connection between the particular nature of child-parent relations in adolescence and the hierarchy of communication needs of adolescents in communication with their parents in the online and offline formats.

The authorial QANCP should be tested in further research. For a deeper study of the phenomenon of communication between children and parents on the Internet, we will resort to qualitative research in the future.

## Limitations

The development of emotional autonomy in the child-parent relationship is an important part of adolescent psychological development. The expansion of the Russian methods of emotional autonomy measure will open to scientists a way to research this important modern formation.

One limitation of the study was the sample size. One of the ways to improve the validity is to expand the sample in follow-up research.

Another limitation was the absence of a factor analysis and a correct test of structural validity, which is due to the structure of the questionnaire and the objectives of the current study. However, conducting these statistical tests becomes an important task for further research.

Another limitation of the study was the nature of the sample used: students from several schools in Moscow, a megalopolis city. We consider participation in the study of students from more schools in Moscow, as well as their peers from cities and settlements from other regions (not only megacities) as an important task of further research.

Another limitation is the acceptable, but not perfect fit indices. The factor model can be reviewed considering the features of Russian sample.

## Appendix

***“Questionnaire of adolescents’ needs in communication with their parents online and o$ine (original version in Russian)***

Опросник «Потребности подростков в общении с родителями «онлайн» и «офлайн»

Инструкция ::

Ниже приведены высказывания, описывающие ваше общение с родителями в реальной жизни. Пожалуйста, прочитайте каждое высказывание и выберите насколько справедливо оно для Вас.

(Below are statements describing your communication with your parents in real life. Please read each statement and choose how fair it is to you.)

1. — нет (no)
2. — скорее нет (probably not)
3. — и да, и нет (yes and no)
4. — скорее да (probably yes)
5. — да (yes)

**Table:**

№	Утверждение	Оценка				
1.	Я общаюсь с родителями потому, что мне важно не по-терять связь с ними *(I communicate with my parents because it is important for me not to lose touch with them)*	1	2	3	4	5
2.	Я общаюсь с родителями, чтобы чувствовать себя частью своей семьи *(I communicate with my parents to feel like a part of my family)*	1	2	3	4	5
3.	Я общаюсь с родителями, чтобы получить их сопережи-вание *(I communicate with my parents to get their empathy)*	1	2	3	4	5
4.	Я общаюсь с родителями, так как мне важно чувствовать их заботу *(I communicate with my parents because it is important for me to feel their care)*	1	2	3	4	5
5.	Я общаюсь с родителями, так как они нуждаются в моей помощи и поддержке *(I communicate with my parents because they need my help and support)*	1	2	3	4	5
6.	Я общаюсь с родителями для решения вопросов совмест-ного быта *(I communicate with my parents to resolve issues of living together)*	1	2	3	4	5
7.	Я общаюсь с родителями для получения новых знаний и опыта *(I communicate with my parents to gain new knowledge and experience)*	1	2	3	4	5
8.	Я общаюсь с родителями, чтобы получить их оценку своих действий *(I communicate with my parents to get their assessment of my actions)*	1	2	3	4	5
9.	Я общаюсь с родителями, чтобы обсудить вопросы миро-воззрения и морали *(I communicate with my parents to discuss issues of worldview and morality)*	1	2	3	4	5
10.	Я общаюсь с родителями для того, чтобы урегулировать взаимоотношения с ними *(I communicate with my parents in order to settle the relation-ship with them)*	1	2	3	4	5
11.	Я общаюсь с родителями, чтобы чувствовать свою при-надлежность к семье *(I communicate with my parents to feel like I belong to the family)*	1	2	3	4	5
12.	Я общаюсь с родителями, так как нуждаюсь в их сочув-ствии *(I communicate with my parents because I need their sympathy)*	1	2	3	4	5
13.	Я общаюсь с родителями, так как нуждаюсь в их помощи и поддержке *(I communicate with my parents because I need their help and support)*	1	2	3	4	5
14.	Я общаюсь с родителями, так как им важно чувствовать мою заботу *(I communicate with my parents because it is important for them to feel my care)*	1	2	3	4	5
15.	Я общаюсь с родителями для выполнения наших совмест-ных дел *(I communicate with my parents to carry out our joint tasks)*	1	2	3	4	5
16.	Я делюсь с матерью / отцом своими проблемами и пере-живаниями *(I share my problems and worries with my mother / father)*	1	2	3	4	5
17.	Я общаюсь с родителями для того, чтобы поделиться с ними своими знаниями и опытом *(I communicate with my parents in order to share my knowledge and experience with them)*	1	2	3	4	5
18.	Я общаюсь с родителями, чтобы обсудить актуальные со-бытия внешнего мира *(I communicate with my parents to discuss current events in the outside world)*	1	2	3	4	5

Ниже приведены высказывания, описывающие ваше общение с родителями в интернет-пространстве. Пожалуйста, прочитайте каждое высказывание и выберите насколько справедливо оно для Вас

(Below are statements describing your communication with your parents in the Internet space. Please read each statement and choose how fair it is to you)

1. — нет (no)
2. — скорее нет (probably not)
3. — и да, и нет (yes and no)
4. — скорее да (probably yes)
5. — да (yes)

**Table:**

№	Утверждение	Оценка				
1.	Я общаюсь с родителями в интернете потому, что мне важно не потерять связь с ними *(I communicate with my parents online because it is important for me not to lose touch with them)*	1	2	3	4	5
2.	Я общаюсь с родителями в интернете, чтобы чувствовать себя частью своей семьи *(I communicate with my parents online to feel like a part of my family)*	1	2	3	4	5
3.	Я общаюсь с родителями в интернете, чтобы получить их сопереживание *(I communicate with my parents online to get their empathy)*	1	2	3	4	5
4.	Я общаюсь с родителями в интернете, так как мне важно чувствовать их заботу *(I communicate with my parents online, as it is important for me to feel their care)*	1	2	3	4	5
5.	Я общаюсь с родителями в интернете, так как они нуждаются в моей помощи и поддержке *(I communicate with my parents online, as they need my help and support)*	1	2	3	4	5
6.	Я общаюсь с родителями в интернете для решения вопро-сов совместного быта *(I communicate with my parents on the Internet to resolve issues of living together)*	1	2	3	4	5
7.	Я общаюсь с родителями в интернете для получения новых знаний и опыта *(I communicate with my parents online to gain new knowledge and experience)*	1	2	3	4	5
8.	Я общаюсь с родителями в интернете, чтобы получить их оценку своих действий *(I communicate with my parents online to get their assessment of my actions)*	1	2	3	4	5
9.	Я общаюсь с родителями в интернете, чтобы обсудить вопросы мировоззрения и морали *(I communicate with my parents online to discuss issues of worldview and morality)*	1	2	3	4	5
10.	Я общаюсь с родителями в интернете для того, чтобы урегулировать взаимоотношения с ними *(I communicate with my parents on the Internet in order to settle the relationship with them)*	1	2	3	4	5
11.	Я общаюсь с родителями в интернете, чтобы чувствовать свою принадлежность к семье *(I communicate with my parents online to feel like I belong to the family)*	1	2	3	4	5
12.	Я общаюсь с родителями в интернете, так как нуждаюсь в их сочувствии *(I communicate with my parents online because I need their sympathy)*	1	2	3	4	5
13.	Я общаюсь с родителями в интернете, так как нуждаюсь в их помощи и поддержке *(I communicate with my parents online because I need their help and support)*	1	2	3	4	5
14.	Я общаюсь с родителями в интернете, так как им важно чувствовать мою заботу *(I communicate with my parents online, as it is important for them to feel my care)*	1	2	3	4	5
15.	Я общаюсь с родителями в интернете для выполнения наших совместных дел *(I communicate with my parents on the Internet to carry out our joint tasks)*	1	2	3	4	5
16.	Я делюсь с матерью своими проблемами и переживаниями *(I share my problems and worries with my mother)*	1	2	3	4	5
17.	Я общаюсь с родителями в интернете для того, чтобы по-делиться с ними своими знаниями и опытом *(I communicate with my parents online in order to share my knowledge and experience with them)*	1	2	3	4	5
18.	Я общаюсь с родителями в интернете, чтобы обсудить актуальные события внешнего мира *(I communicate with my parents online to discuss current events in the outside world)*	1	2	3	4	5
